# Severe Hypocalcemia and Resulting Seizure Caused by Vitamin D Deficiency in an Older Patient Receiving Phenytoin: Eldecalcitol and Maxacalcitol Ointment as Potential Therapeutic Options for Hypocalcemia

**DOI:** 10.1155/2019/3653276

**Published:** 2019-10-15

**Authors:** Seishi Aihara, Shunsuke Yamada, Mika Kondo, Hideaki Oka, Taro Kamimura, Atsumi Harada, Toshiaki Nakano, Kazuhiko Tsuruya, Takanari Kitazono

**Affiliations:** ^1^Kidney Center, Matsuyama Red Cross Hospital, Japan; ^2^Department of Medicine and Clinical Science, Graduate School of Medical Sciences, Kyushu University, Fukuoka, Japan; ^3^Department of Nephrology, Nara Medical University, Nara, Japan

## Abstract

An 82-year-old man treated with phenytoin for the prevention of symptomatic epilepsy was hospitalized to treat consciousness disturbance, seizure, and hypocalcemia (serum calcium: 4.6 mg/dL). Serum 25-hydroxyvitamin D level was very low (5.4 ng/mL), whereas serum calcitriol level was normal (27 pg/mL) and serum intact parathyroid hormone level was increased (369 pg/mL). He was finally diagnosed with vitamin D deficiency associated with low sunlight exposure and long-term phenytoin use for symptomatic epilepsy: phenytoin is shown to accelerate catabolism of 25-hydroxyvitamin D. Combination treatment with eldecalcitol and maxacalcitol ointments successfully normalized corrected serum calcium level: both eldecalcitol and maxacalcitol are vitamin D receptor activators used for osteoporosis and psoriasis, respectively. Our case illustrates the importance of periodic serum calcium level monitoring in patients receiving anti-epileptic drugs and the usefulness of eldecalcitol and maxacalcitol ointment as a therapeutic option for hypocalcemia, especially in countries where native vitamin D and 25-hydroxyvitamin D are not available.

## 1. Background

Hypocalcemia is a lethal complication that can lead to arrhythmia, heart failure, and seizure [1–3]. Serum calcium (Ca) concentrations are normally maintained within a very narrow range that is required for optimal extracellular and intracellular function [4]. The major factors determining serum Ca concentration are parathyroid hormone (PTH), calcitriol, ionized Ca itself, vitamin D, and serum phosphate level [5]. Low serum Ca concentration, hypocalcemia, is most often caused by disordered regulation and metabolism of PTH and vitamin D axis [6]. For example, among medical conditions not caused by genetic disorders, primary hypoparathyroidism and vitamin D deficiency with or without chronic kidney disease (CKD) are the two major causes of hypocalcemia; patients with advanced CKD often have low serum calcitriol level, which is mediated by increased fibroblast growth factor 23 [7, 8].

Another factor related to vitamin D activity is disordered vitamin D metabolism associated with altered cytochrome P-450 activity [9]. A variety of drugs can affect cytochrome P-450 activity, which is a key enzyme metabolizing endogenous and exogenous substances in the human body. In vitamin D metabolism, anti-epileptic drugs (AEDs), including phenytoin, are known to interact with cytochrome P-450 activity. AEDs accelerate the rate of 25-hydroxyvitamin D and 1*α*-25-dihydroxyvitamin D catabolism to inactive metabolites, occasionally leading to vitamin D deficiency and hypocalcemia [10].

We present a case of an older patient with seizure and very severe hypocalcemia caused by vitamin D deficiency, which was likely associated with long-term use of AEDs and lack of sunlight exposure, who was successfully treated with combined treatment with oral Ca supplement and vitamin D receptor activators (VDRAs), eldecalcitol, and maxacalcitol ointments. Our case also suggests the potential usefulness of eldecalcitol and maxacalcitol ointment in the management of hypocalcemia, especially in countries where native vitamin D or 25-hydroxyvitmin D is not available for the treatment of vitamin D deficiency-induced hypocalcemia.

## 2. Case Presentation

An 82-year-old man was hospitalized because of fever, seizure, and consciousness disturbance. He had a history of gastrectomy for gastric ulcer when he was young. At age 74 years, he developed subarachnoid hemorrhage and secondary symptomatic epilepsy, and was treated with phenytoin, an AED. With progressive disuse syndrome after the brain event, he had been bedridden for the last 8 years under insufficient sunlight exposure. Two weeks before hospitalization, he suffered an acute seizure, followed by consciousness disturbance. Thereafter, food intake and oral dosing were insufficient. Three days before hospitalization, he developed a fever >38°C. Because of concurrent respiratory failure, he was transferred to our hospital for further treatment.

On admission, the patient showed impaired consciousness with closed eyes. Verbal response was incomprehensible, and the best motor response was a response to localized pain (Glasgow coma scale score: E1V2M5). His vital signs were: blood pressure, 84/52 mmHg; heart rate, 84 bpm; body temperature, 40.0°C; and arterial blood oxygen saturation measured by pulse oximetry of 94% with supplemental oxygen at 8 L/min. His height was 161 cm, his weight was 48.5 kg, and his body mass index was 18.7 kg/m^2^. He showed neither jolt accentuation of headache nor spastic rigidity. A coarse crackle was heard in his left lung. Importantly, Chvostek and Trousseau signs indicative of tetany were positive.

Laboratory data on admission are shown in Table 1. Briefly, the following results were obtained: white blood cell count (10 390/*μ*L), serum albumin (3.1 g/dL), blood urea nitrogen (13.8 mg/dL), creatinine (0.44 mg/dL), estimated glomerular filtration rate (134.5 mL/min/1.73 m^2^), phosphate (1.6 mg/dL), Ca (3.7 mg/dL), ionized Ca (0.55 mmol/L), magnesium (2.97 mg/dL), intact PTH (386.8 pg/mL), and serum C-reactive protein (1.67 mg/dL). Although blood phenytoin concentration was low (<0.8 *µ*g/mL), this value was plausible because phenytoin has a half-life of 20–30 h, and it was not being administered sufficiently at the time of hospitalization over the last few days. An electrocardiogram showed a sinus rate of 93 beats per minute with long QT interval (0.398 s) and corrected QT-T interval (0.398 second).

**Figure 1:**
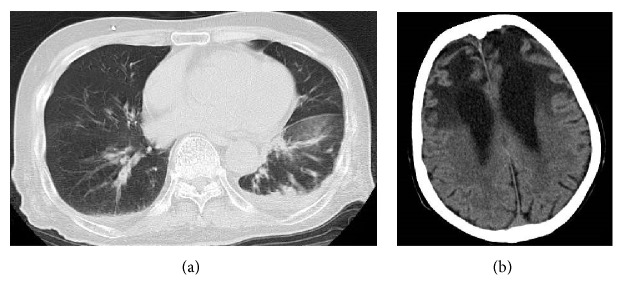
Plain nonenhanced computed tomographic images of the lung and head. (a) Image shows a consolidation suspected as pneumonia at the bottom of the patient's left lung with concurrent interlobar pleural effusion and pleural effusion. (b) Although acute-phase lesions such as bleeding were not seen, frontal lobe atrophy and ventricular enlargement were confirmed.

At the bottom of his left lung, a consolidation was visible that was suspected pneumonia, and interlobar pleural effusion was also identified on non-contrast-enhanced computed tomography (Figure 1(a)). Although acute-phase lesions such as bleeding were not observed, frontal lobe atrophy and ventricular enlargement were confirmed by head computed tomography (Figure 1(b)). Based on these results, he was diagnosed as having bacterial pneumonia and its related sepsis. Because the patient showed neither jolt accentuation of headache nor spastic rigidity, meningitis was ruled out as a cause of seizure.

At that point, several differential diagnoses were considered for the hypocalcemia. The serum Ca level corrected for serum albumin was 4.6 mg/dL, and ionized Ca was 0.55 mmol/L, indicating true hypocalcemia, not pseudo-hypocalcemia. CKD was also excluded as a possible cause based on his kidney function. There was also no evidence of severe hypomagnesemia to cause decreased secretion of PTH, although serum magnesium level was slightly low (1.6 mg/dL). In addition, serum intact PTH level on admission was very high. Accordingly, we considered that parathyroid gland was normally functioning because intact PTH appropriately increased in response to hypocalcemia. Bone formation was slightly increased in response to increased serum intact PTH, whereas bone resorption was decreased judging from the serum tartrate-resistant acid phosphatase-5b level. At that point, the differential diagnosis of hypocalcemia still included pancreatitis and sepsis. Pancreatitis was ruled out based on the normal serum amylase and lipase levels and absence of pancreas enlargement by imaging. Although the mechanism of hypocalcemia in septic patients is complex and not well understood, previous studies showed that kidneys are reported to increase urinary Ca excretion in sepsis [11]. However, fractional excretion of Ca was remarkably low (0.13%), indicating Ca resorption enhancement across renal tubules. Taken together, sepsis-related hypocalcemia was not regarded as the primary cause of the hypocalcemia in the present case.

Another potential mechanism for the severe hypocalcemia was decreased Ca absorption from the gastrointestinal tract. Because he had been in a nursing home, which was an environment with extremely low sunlight exposure, vitamin D_3_ generally produced in the skin was potentially lacking. Notably, he was on long-term treatment with phenytoin, which is shown to accelerate conversion of 25-hydroxyvitamin D to 24,25-dihydroxyvitamin D [9, 12]. The low level of 25-hydroxyvitamin D (5.4 ng/mL), which is an indicator of vitamin D status in the body, strongly indicated that he had been suffering from 25-hydroxyvitamin D deficiency, probably caused by combination of low sunlight exposure and long-term use of phenytoin.

**Figure 2:**
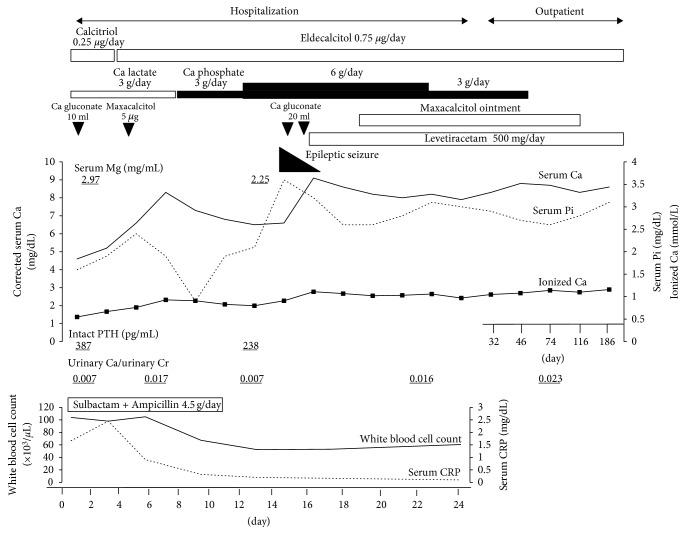
Patient's clinical course before and after the combined medical treatment. Ca, calcium; Cr, creatinine; CRP, C-reactive protein; PTH, parathyroid hormone; Pi, phosphate; Mg, magnesium.

The patient's clinical course is shown in Figure 2. He received intravenous hydration for sepsis and antibiotics to target anaerobes (sulbactam + ampicillin 4.5 g/day) for possible aspiration pneumonia. He was treated with intravenous Ca gluconate (8.5% 10–20 mL, 3.9–7.8 mEq/day) and trans-gastric tube administration of Ca lactate/phosphate hydrate (3–6 g/day) and we discontinued phenytoin from the first hospital day. Administration of 0.25 *µ*g/day of calcitriol by nasal gastric tube was performed between the first and second hospital days. From the third hospital day, we exchanged calcitriol with 0.75 *µ*g/day of eldecalcitol, a VDRA often used for osteoporosis in Japan [13], because eldecalcitol is shown to have a higher binding ability to vitamin D binding protein (DBP) than calcitriol: binding ability of eldecalcitol to DBP is 4.4 times higher than that of calcitriol [14]. Indeed, previous studies have shown that vitamin D analogues that have a higher affinity for DBP show a longer half-life. Although the mechanism remains unclear, vitamin D analogue that binds tightly to the DBP may be less catalyzed by the cellular 24-hydroxylase activity, which is expressed in the target cells of vitamin D [15]. After treatment with effective antibiotics, inflammatory markers decreased steadily, and oxygen support was discontinued. His consciousness recovered after improvements in the pneumonia and hypocalcemia. Glasgow coma scale score on the 8th hospital day was E4V4M6, and he was able to eat orally. Intravenous Ca gluconate was continued until his serum Ca level increased to within the reference range. While continuing intravenous Ca gluconate and oral administration of calcitriol and Ca supplement, we administered maxacalcitol ointment, another VDRA used for psoriasis. Maxacalcitol was applied to trunk and limbs twice daily with less than 10 g/day. The absorption rate, maximum drug concentration time, and biological half-life of maxacalcitol ointment are 83.1%, 3 h, and 2–4 h, respectively. An epileptic seizure occurred on the 15th hospital day when his serum Ca level was still below the lower reference limit. The seizure episode might also have been caused by his prolonged hypocalcemia or might have been related to his history of traumatic subarachnoid hemorrhage. Thereafter, oral administration of levetiracetam was started.

After those combined treatment, his serum corrected Ca level finally increased up to 8.2 mg/dL, and his serum phosphate level increased from 1.6 mg/dL to 3.1 mg/dL. After his serum Ca and phosphate levels were stabilized, he was discharged on the 24th hospital day. At 3 months after discharge, he was in the nursing home without relapse of hypocalcemia, followed by discontinuation of maxacalcitol ointment. With continuous eldecalcitol administration only, his serum Ca level remained stable after 5 months.

## 3. Discussion

In the present case, we successfully treated vitamin D deficiency-induced hypocalcemia and seizure associated with phenytoin use and lack of sunlight exposure in an older patient without CKD, who had a history of traumatic subarachnoidal hemorrhage. Our treatment protocol included administering Ca preparations, oral eldecalcitol, and maxacalcitol ointments, and discontinuation of phenytoin. After 24 days of these combined treatments, the patient's serum Ca level normalized, and no further seizures occurred.

Hypocalcemia has many causes and results primarily from inadequate PTH secretion or PTH resistance, vitamin D deficiency or vitamin D resistance, abnormal magnesium metabolism, and extravascular deposition of Ca salts, which can occur with several medical conditions [16, 17]. In our patient, serum levels of PTH and alkaline phosphatase were elevated and fractional excretion of Ca was very low, while serum levels of magnesium and calcitriol were within the reference ranges, indicating that the parathyroid gland was functioning normally in response to the hypocalcemia, and that the kidneys were acting to enhance tubular Ca reabsorption. Bone formation was slightly increased in response to increased serum intact PTH, whereas bone resorption was decreased judging from the serum tartrate-resistant acid phosphatase-5b. Importantly, serum 25-hydroxyvitamin D level was extremely low, indicating that 25-hydroxyvitamin D deficiency was the primary cause of the hypocalcemia in our patient, and that all other changes in the serum and urine mineral markers were secondary.

There were two possible reasons for the 25-hydroxyvitamin D deficiency. One is the long-term use of phenytoin to prevent seizure secondary to the previous traumatic subarachnoid hemorrhage, because AEDs including phenytoin accelerate 24-hydroxylase activity and thereby decrease serum 25-hydroxyvitamin D level, inducing hypocalcemia [18, 19]. There are no past reports about other effects of AEDs on Ca metabolism; however, AEDs may have other potential influence on Ca metabolism, such as decreasing Ca absorption from the gastrointestinal tract or reducing cellular sensitivity to PTH in the parathyroid gland. Another factor affecting the low serum calcitriol level might have been that our patient was bedridden and had less opportunity for sunlight exposure; 25-hydroxyvitamin D is partially derived from vitamin D_3_ produced in the skin in response to sunlight exposure. Because older patients with histories of stroke or other brain disorders are often bedridden and treated with AEDs to prevent symptomatic epilepsy, these patients are at increased risk of 25-hydroxyvitamin D deficiency-induced hypocalcemia. Because today's population is aging rapidly, leading to increased numbers of older people in nursing homes who are experiencing decreased activity, the chances of a similar clinical scenario are also increased in clinical practice. Our case highlights the importance of periodic monitoring of serum Ca levels to prevent the severe hypocalcemia induced by vitamin D deficiency.

Treatment strategies for hypocalcemia differ depending on the severity and mode of onset of hypocalcemia. As a replacement therapy for vitamin D deficiency, we selected eldecalcitol, which has a higher binding ability to vitamin D binding protein (DBP) [13, 14]. Because non-protein-bound vitamin D is easily catabolized locally or in the liver, complex of 25-hydroxyvitamin D and DBP is resistant to degradation. Generally, eldecalcitol is usually used for osteoporosis and potentially increases serum Ca level; 0.88% of patients treated with eldecalcitol for osteoporosis developed hypercalcemia during the course of treatment [20]. Actually, based on our previous study, we think that patients treated with eldecalcitol are likely to develop hypercalcemia and acute kidney injury more frequently than has been reported, especially female patients with lower body weight [21]. Therefore, eldecalcitol may be more useful than calcitriol when treating patients with hypocalcemia. Using concurrent maxacalcitol ointment, we successfully controlled serum Ca levels within the reference range. As for maxacalcitol ointment, hypercalcemia is a potential side effect because it is absorbed through skin and increases Ca absorption from the gastrointestinal tract [22]. In this regard, this maxacalcitol-related hypercalcemia may be wisely used to treat hypocalcemia, in some special conditions where oral administration of native vitamin D or 25-hydroxyvitamin D is not an option.

Our treatment strategy to treat vitamin D deficiency-induced hypocalcemia with VDRAs may not be accepted by some physicians. Actually, phenytoin-induced hypocalcemia should be treated with native vitamin D or 25-hydroxyvitamin D administration and discontinuation of phenytoin. However, both native vitamin D and 25-hydroxyvitamin D are not available as official drugs in Japan: only native vitamin D is available as supplement. Accordingly, we administered VDRAs instead of native vitamin D or 25-hydroxyvitamin D. In this regard, our strategy to use VDRAs for vitamin D deficiency-induced hypocalcemia could be justified in countries where native vitamin D and 25-hydroyvitamin D are not available. We also warn against the potential danger that VDRAs can induce hypercalcemia and hyperphosphatemia and should be cautiously used with a close monitoring of serum levels of Ca and phosphate.

Hypocalcemia might have contributed to the seizure that occurred 2 weeks prior to hospitalization and/or on the 15th hospital day. Unfortunately, the patient was in a nursing home at 2 weeks prior to the hospitalization and his attending physician did not examine his serum Ca level. Therefore, we were unable to know his serum Ca levels before hospitalization. This is a limitation of the present case. Importantly, hypocalcemia can cause seizures without concomitant tetany because low ionized Ca concentrations in the cerebrospinal fluid are associated with increased excitability in the central nervous system [2, 3]. In our case, vitamin D deficiency was probably a chronic condition due to the long history of phenytoin use and lack of sun exposure. Hence, we thought that there must have been some factors that triggered hypocalcemia-induced seizure. It is plausible that his excitability of the motor area was enhanced by superimposing metabolic factors such as infection-related fever on the vulnerable cerebral cortex, ultimately leading to the seizure episodes. Importantly, patients with a history of symptomatic epilepsy commonly receive AEDs and are at increased risk of AED-associated hypocalcemia, which may in turn lower the seizure threshold. Therefore, we must be aware that vitamin D deficiency-induced hypocalcemia could be caused by AEDs and consider this an old but very important differential diagnosis, especially when we encounter hypocalcemic older patients receiving AEDs.

In summary, we presented a case of severe symptomatic hypocalcemia caused by vitamin D deficiency, which was likely associated with the long-term use of phenytoin and lack of sunlight exposure in a nonCKD patient. Although the best strategy to treat phenytoin-induced hypocalcemia would be treatment with native vitamin D replacement and discontinuation of phenytoin, it is a treatment option to administer vitamin D receptor activators such as eldecalcitol and maxacalcitol ointment for severe hypocalcemic patients, especially in countries where native vitamin D or 25-hydroxyvitamin D is not available as treatment option. Besides, periodic monitoring of serum Ca levels is necessary to prevent severe symptomatic hypocalcemia induced by vitamin D deficiency in patients treated with AEDs.

## Figures and Tables

**Table 1:** Patient's laboratory data on admission.

		Reference range
*Complete blood count*		
White blood cells	10 390/μL	
Neutrophils	90.6%	
Red blood cells × 10^4^	342/*μ*L	
Hemoglobin	10.8 g/dL	
Platelets × 10^4^	26.5/*μ*L	

*Serum biochemistry*		
Total protein	6.2 g/dL	
Albumin	3.1 g/dL	
Blood urea nitrogen	13.8 mg/dL	
Creatinine	0.44 mg/dL	
Uric acid	1.9 mg/dL	
Total bilirubin	0.3 mg/dL	
Aspartate aminotransferase	74 U/L	
Alanine aminotransferase	36 U/L	
Alkaline phosphatase	463 U/L	
*γ*‐Glutamyl transpeptidase	33 U/L	
Lactate dehydrogenase	729 U/L	
Total cholesterol	186 mg/dL	
Amylase	158 U/L	
LDL‐cholesterol	96 mg/dL	
Sodium	131 mEq/L	
Potassium	3.1 mEq/L	
Chloride	92 mEq/L	
Calcium	3.7 mg/dL	
Phosphate	1.6 mg/dL	
Magnesium	2.97 mg/dL	
Hemoglobin A1c (NGSP)	5.6%	

*Coagulation*		
PT‐INR	1.20	
APTT	32.1 sec	

*Immunological testing*		
C‐reactive protein	1.67 mg/dL	
Procalcitonin	0.52 mg/dL	

*Endocrinology*		
Intact PTH	386.8 pg/mL	10–65
Calcitriol	27 pg/mL	20–60
25‐hydroxyvitamin D	5.4 ng/mL	9.0–33.9
TRACP‐5b	116 mU/mL	120–420
BAP	31.3 *μ*g/L	3.8–22.6
Osteocalcin	10.2 ng/mL	*N*; 2.5–13
Calcitonin	<0.5 pg/mL	26.2–49.0

*Arterial blood gas*		
pH	7.458	
PaO_2_	81.7 mmHg	
PaCO_2_	33.9 mmHg	
HCO_3_^−^	23.7 mmol/L	
Ca^2+^	0.55 mmol/L	

*Urinary biochemistry*		
Urinary creatinine	121.7 mg/dL	
Urinary calcium	0.8 mg/dL	
Urinary phosphate	22.6 mg/dL	

Abbreviations: APTT, activated partial thrombin time; BAP, bone‐type alkaline phosphatase; Ca^2+^, ionized calcium; HCO_3_^−^, hydrogen carbonate; LDL, low‐density lipoprotein; NGSP, national glycohemoglobin standardization program; PaCO_2_, partial pressure of arterial carbon dioxide; PaO_2_, partial pressure of arterial oxygen; PT‐INR, prothrombin time‐international normalized ratio; PTH, parathyroid hormone; TRACP‐5b, tartrate‐resistant acid phosphatase 5b.

## Data Availability

The data used to support the findings of this study are available from the corresponding upon request.
